# Dynamic gating window technique for the reduction of dosimetric error in respiratory‐gated spot‐scanning particle therapy: An initial phantom study using patient tumor trajectory data

**DOI:** 10.1002/acm2.12832

**Published:** 2020-02-18

**Authors:** Naoki Miyamoto, Kouhei Yokokawa, Seishin Takao, Taeko Matsuura, Sodai Tanaka, Shinichi Shimizu, Hiroki Shirato, Kikuo Umegaki

**Affiliations:** ^1^ Division of Quantum Science and Engineering Faculty of Engineering Hokkaido University Sapporo Japan; ^2^ Global Station for Quantum Medical Science and Engineering Global Institution for Collaborative Research and Education (GI‐CoRE) Hokkaido University Sapporo Japan; ^3^ Department of Medical Physics Hokkaido University Hospital Sapporo Japan; ^4^ Department of Radiation Medical Science and Engineering Faculty of Medicine Hokkaido University Sapporo Japan; ^5^ Department of Radiation Medicine Faculty of Medicine Hokkaido University Sapporo Japan

**Keywords:** gated irradiation, gating window, proton therapy, respiratory motion, spot‐scanning

## Abstract

Spot‐scanning particle therapy possesses advantages, such as high conformity to the target and efficient energy utilization compared with those of the passive scattering irradiation technique. However, this irradiation technique is sensitive to target motion. In the current clinical situation, some motion management techniques, such as respiratory‐gated irradiation, which uses an external or internal surrogate, have been clinically applied. In surrogate‐based gating, the size of the gating window is fixed during the treatment in the current treatment system. In this study, we propose a dynamic gating window technique, which optimizes the size of gating window for each spot by considering a possible dosimetric error. The effectiveness of the dynamic gating window technique was evaluated by simulating irradiation using a moving target in a water phantom. In dosimetric characteristics comparison, the dynamic gating window technique exhibited better performance in all evaluation volumes with different effective depths compared with that of the fixed gate approach. The variation of dosimetric characteristics according to the target depth was small in dynamic gate compared to fixed gate. These results suggest that the dynamic gating window technique can maintain an acceptable dose distribution regardless of the target depth. The overall gating efficiency of the dynamic gate was approximately equal or greater than that of the fixed gating window. In dynamic gate, as the target depth becomes shallower, the gating efficiency will be reduced, although dosimetric characteristics will be maintained regardless of the target depth. The results of this study suggest that the proposed gating technique may potentially improve the dose distribution. However, additional evaluations should be undertaken in the future to determine clinical applicability by assuming the specifications of the treatment system and clinical situation.

## 1 INTRODUCTION

The number of proton therapy systems that implement the spot‐scanning irradiation technique has been increasing in recent years worldwide. The spot‐scanning technique possesses advantages including high conformity to the target and efficient energy utilization compared with those of the passive scattering irradiation technique. In spot‐scanning technique, all or most protons will be delivered to the patients, although many protons are blocked/collimated out in the passive scattering technique. In addition, spot‐scanning enables intensity‐modulated particle therapy because the intensity (MU, time or fluence) of each spot can be easily modulated.^1^, ^2^ However, this irradiation technique is sensitive to target motion. Because the dose is delivered spot‐by‐spot, local under‐ and over‐dosages inside the target volume can be created owing to the interplay effect between the pencil beam and tumor motion. Thus, management of respiratory motion of the tumor is essential especially for the spot‐scanning particle therapy. Various motion management techniques, which use an external surrogate^3^, ^4^, ^5^, ^6^, ^7^, ^8^, ^9^, ^10^ or an internal fiducial marker,^11^, ^12^ have been clinically applied. External surrogates, such as monitoring of the abdominal motion with a laser displacement sensor, are used to obtain a respiratory signal. Thus, the treatment beam is gated only when the respiratory signal is within the predefined region, which is called gating window. However, external motion is not necessarily correlated with internal motion during treatment.^13^, ^14^, ^15^ For example, it has been reported that the frequency of baseline shift/drift can increase with longer treatment time.^16^ To realize beam gating with internal target motion monitoring, real‐time image gated proton therapy (RGPT) has been developed.^11^, ^12^ In RGPT, as in real‐time tumor‐tracking therapy (RTRT) during photon therapy,^17^, ^18^ two orthogonal fluoroscopic images are continuously acquired during the treatment, and a three‐dimensional position of the internal fiducial marker, which is inserted in or near the tumor is obtained in real‐time. The treatment beam irradiates only when the fiducial marker is within the three‐dimensional region, i.e., the gating window. It has been reported that clinically acceptable dose can be delivered with a fixed gate of ±2 mm during RGPT.^11^, ^19^ During this treatment technique, patient position can be corrected if the baseline shift has occurred.^20^ In both external and internal surrogate‐based gating, the size of the gating window is fixed during the treatment in the current treatment system. Dosimetric error can be reduced more by decreasing the size of the gating window. However, treatment time can be prolonged because the gating efficiency is lower.

Total dose distribution is constituted from the spots for which the doses are different during spot‐scanning particle therapy. For example, by assuming that the spread‐out Bragg peak (SOBP) is constructed from multiple Bragg peaks, the dosimetric error in a distal layer will be large compared with that in a proximal layer for the same positional error of the spot. This means that the size of the gating window can be optimized for each spot. In this study, we propose the dynamic gating window technique, which optimizes the size of the gating window for each spot by considering the possible dosimetric error. Although a treatment system with a dynamic gating window technique function is not available for clinical applications, the implementation of this function can be addressed by software/hardware modifications, as described in the discussion section. The purpose of this study is to show the effectiveness of the dynamic gating window technique by evaluating the dosimetric error and the gating efficiency by simulating irradiation of the moving target in a water phantom. As an initial study, the target was assumed to be a cubic region. Target motion was simulated using the actual three‐dimensional trajectory data, which were obtained during lung RTRT.

## 2 MATERIALS AND METHODS

### 2.1 Dynamic gating window technique

**Figure 1:**
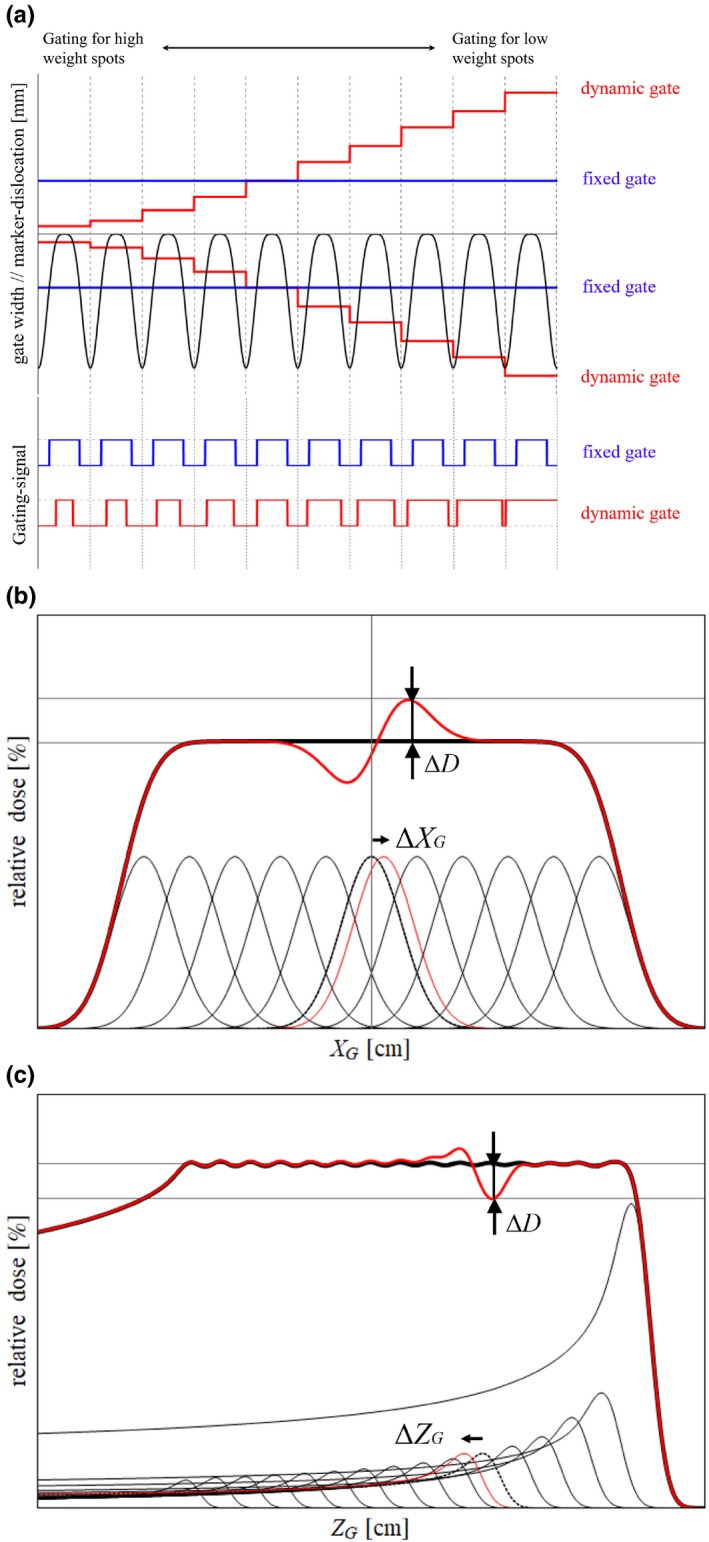
(a) Schematic diagram of the marker trajectory, size of the gating window for dynamic and fixed methods, and the corresponding gate signal pattern. The relationship between the deviation of the spot position and possible dosimetric error in the (b) scanning and (c) beam directions.

The concept of the dynamic gating window technique is shown in Fig. 1. The region of the gating window is defined in gantry coordinates. Specifically, *X*_G_ and *Y*_G_ correspond to the scan direction, and *Z*_G_ corresponds to the beam direction. In the proposed technique, the size of the gating window changes for each spot during the treatment to suppress the dosimetric error within a predefined tolerance level as shown in Fig. 1(a). Specifically, the smaller size of the gating window is used for the irradiation in a distal layer where the large dosimetric error can occur. In contrast, the larger size of the gating window is used in a proximal layer. Thus, gating efficiency can be varied even if the target motion remains constant. By evaluating the possible dosimetric error ΔD owing to the deviation of the irradiated spot position Δ*X*_G_, Δ*Y*_G_, and Δ*Z*_G_, as shown in Figs. 1(b) and 1(c), the size of the gating window can be determined for each direction *X*_G_, *Y*_G_, and *Z*_G_. For simplicity, in this study, Δ*X*_G_, Δ*Y*_G_, and Δ*Z*_G_ were assumed to be identical to the spatial displacement of the fiducial marker between the planned and irradiated positions. Thus, positional deviation of the fiducial marker along *Z*_G_ can be viewed as the variation of water equivalent length (WEL) in the beam path. Furthermore, the WEL variation owing to Δ*X*_G_ and Δ*Y*_G_ was omitted because a simple water phantom was assumed in the dosimetric evaluation. Thus, an appropriate gate size can be determined because Δ*X*_G_, Δ*Y*_G_, and Δ*Z*_G_ can suppress ΔD less than the tolerance level for each spot. In this study, dosimetric evaluation was conducted with the water phantom in order to demonstrate the effectiveness of the dynamic gating window technique in simple condition as an initial study. Note that the above assumptions about WEL variation would not necessarily be applicable to actual clinical situation.

In this study, the tolerance of dosimetric error to determine the size of the gating window for each direction was set to 5%. The minimum size of the gating window in all directions was limited to 1 mm because the size should not be lower than the measurement accuracy of a typical imaging equipment. The maximum size of the gating window in the scan direction was limited to 5 mm to prevent the spot position error from being larger than the spot spacing that is used in the evaluation even if the corresponding dosimetric error was within the tolerance.

### 2.2 Dosimetric simulation

#### 2.2.1 Simulation condition

**Figure 2:**
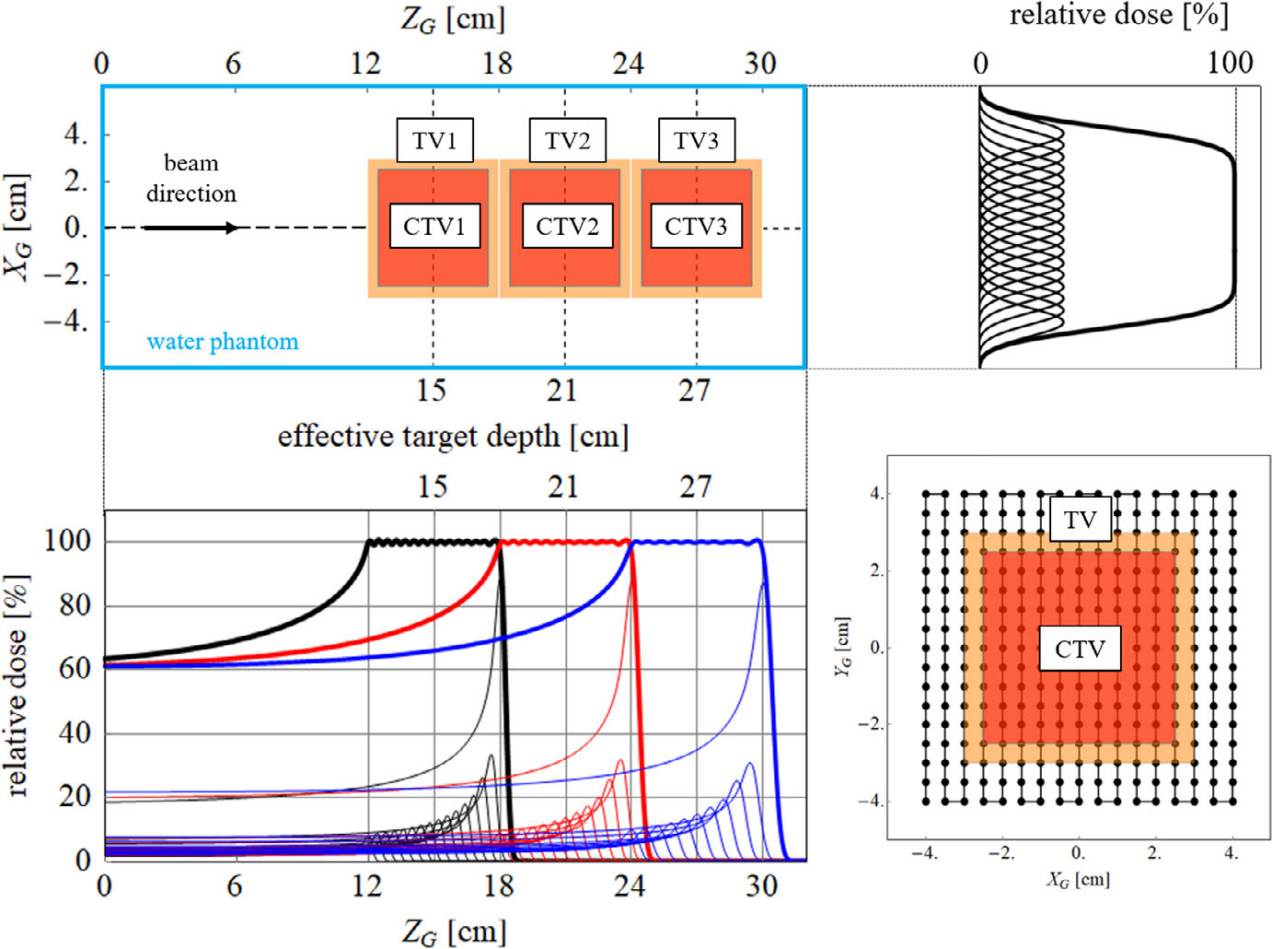
(Left top) Evaluation geometry of each target and a water phantom. An example of dose profile along (right top) the scanning direction and (left bottom) beam direction. (right bottom) Spot positions in each layer.

The effectiveness of the dynamic gating window technique was evaluated by dosimetric simulations performed with a simple mathematical water phantom. The evaluation geometry is shown in Fig. 2. In this study, the treated volume (TV), which is the volume that is planned to receive more than 95% of the prescribed dose, was set to 6 cm^3^ × 6 cm^3^ × 6 cm^3^. It is noted that the TV includes the clinical target volume (CTV) and the margin to consider the uncertainty of dose delivery (e.g., positioning errors and CT number uncertainty). It was assumed that the TV is constituted from a CTV and an isotropic margin of 5 mm. In this study, dosimetric characteristics were evaluated in CTVs and TVs. Because the dose distribution is expected to be improved by the proposed technique, especially in the marginal region on distal side, dosimetric characteristics were also evaluated in the TV to reveal the effectiveness of the proposed technique. Dosimetric characteristics were examined in the CTV and TV with three different depths to evaluate the dependence of layer interval. Effective depth, SOBP range, beam energies, required numbers of layers to create a SOBP, and layer distance for each TV are summarized in Table 1. Effective depth was defined as the depth of the SOBP center. In each case, the TV was moved in the water phantom according to the three‐dimensional trajectory data of lung tumor motion, which was obtained from the photon lung RTRT. The beam direction was anterior to posterior direction. Trajectory data, which included more than 5 mm of beam and scanning directions, were selected to determine the effectiveness of the proposed technique because the dosimetric error is small with or without beam gating for the data with small motion. A total of 34 trajectory data, which were obtained from different patients, were used. Trajectory data were used iteratively in case that the data length was insufficient to irradiate the TV. The three‐dimensional position of the gating window was defined as a location that provides the maximum gating efficiency in the first 20 s of trajectory data for each case because the location of the gating window in the original data was different owing to the manual setup. For comparison, evaluation was also conducted with a fixed gate. The size of gating window was fixed to ±2 mm for all directions, which is a typical setting of RTRT in clinical practice.

**Table 1:** Effective depth, SOBP range, beam energies, required numbers of layers to create an SOBP, and layer distance for each TV.

	TV #1	TV #2	TV #3
Effective depth (cm)	15	21	27
SOBP range (cm)	12–18	18–24	24–30
Energy (MeV)	129.9–163.3	163.3–192.1	192.1–217.9
# of layers	16	13	11
Layer distance (mm)	4	5	6

SOBP, spread‐out Bragg peak; TV, treated volume.

#### 2.2.2 Dose calculation

Dose distribution was calculated using a commercial technical computing tool, Mathematica (Wolfram Research, USA). In this study, a Bragg curve for each spot was modeled by Bortfeld's analytical formula.^21^ For each energy, the beam width, which was measured for the Hokkaido University spot‐scanning proton beam treatment system, was used. Highland approximation was used to consider proton scattering in the water phantom.^22^ The scan started from the most distal layer, and the scan depth was decreased layer by layer until the entire volume was irradiated. Furthermore, 17 × 17 spots for each layer with a spot spacing of 5 mm were used. All spots in a layer had the same dose weight. Thus, the size of the dynamic gate for the spots in a layer was same in this evaluation. In the calculation of dose distribution, spot positions were fixed in all evaluation conditions. The evaluation regions, CTV and TV in this study, were moved in the water phantom according to the motion trajectory of the tumor. The calculation grid was 2 mm for each direction. All evaluations were conducted for one dose painting with a relative dose. Since the derivable maximum MU/spot is varied for each treatment system, the target may need to be irradiated multiple times according to the treatment planning parameters such as prescribed dose. In this study, one‐time irradiation was assumed in order to evaluate the effectiveness of the proposed technique without repaint effect. In addition, dosimetric characteristics were evaluated with relative dose normalized by the maximum value of SOBP because all evaluations were independent of absolute dose.

To simulate beam gating spot‐by‐spot by assuming the actual time scale, the tumor trajectory data, which were recorded at a rate of 30 times per second, were interpolated to evaluate the positional deviation of the target at each irradiation spot. In this study, machine specifications that are similar to those of the typical proton therapy system were assumed. The scan speed was fixed to 10 m/s, which corresponds to 5 ms required to move to the next spot. The spot irradiation time was set to 5 ms per spot for the maximum dose weight. Then, the irradiation time for each spot was evaluated according to the spot weight.

### 2.3 Effectiveness evaluation

#### 2.3.1 Dosimetric characteristics

To evaluate the effectiveness of the dynamic gating window technique, dosimetric characteristics (e.g., maximum dose [*D*_max_], minimum dose [*D*_min_], homogeneity index [HI], and SD in TV) were evaluated for the fixed gate and dynamic gate approaches.

#### 2.3.2 Gating efficiency

In respiratory‐gated radiation therapy, treatment time is one of the main concerns because the treatment time can be prolonged if the gating efficiency is low. It is difficult to evaluate irradiation time quantitatively in respiratory‐gated spot‐scanning particle therapy because it depends on the specifications of the treatment system such as dose rate and particle accelerator used. Thus, in this study, the gating efficiency was evaluated as an index of irradiation time. The gating efficiency was determined as a ratio of the accumulated time of gate‐on to the total time required to finish irradiation. In other words, it was equal to the ratio of the time when the fiducial marker was within the gating window to the time required to finish the irradiation. In this study, the gating efficiency for each irradiation layer was evaluated as a ratio of the gate‐on time to the required time for the irradiation of each layer. The gating efficiency for each layer was averaged for 34 cases. The overall gating efficiency was evaluated as a ratio of the accumulated gate‐on time to the required irradiation time through all the layers. The overall gating efficiency of 34 cases was compared by using box plot.

## 3 RESULTS

### 3.1 Dosimetric characteristics

**Figure 3:**
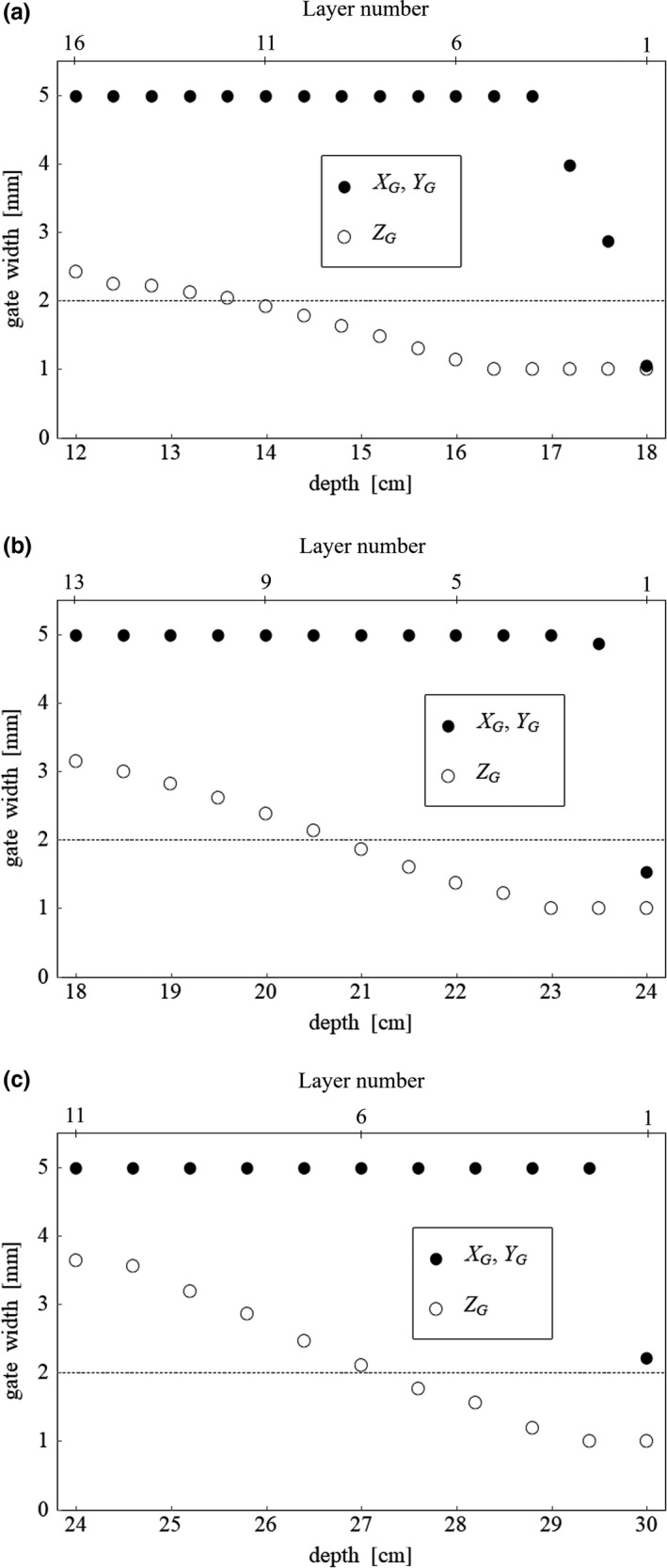
Size of the gating window in the scan direction *X_G_*/*Y_G_* and in the beam direction *Z_G_* for each treated volume (TV) located at effective depths of (a) 15 cm, (b) 21 cm, and (c) 27 cm. Dashed line represents the gate width of 2 mm in the fixed gate.

The sizes of the gating window in *X*_G_, *Y*_G_, and *Z*_G_ directions for the spots in each layer are shown in Fig. 3. In this evaluation, the same size of the gating window was used for the spots in each layer because a simple cubic target was assumed. Regarding the gate size in the beam direction *Z*_G_, the size of the dynamic gate was smaller than that of the fixed gate in the distal region. Because the spot weight decreased under this evaluation condition, the size of the dynamic gate increased in the proximal region. Regarding the size in the scan directions *X*_G_ and *Y*_G_, the window size increased from the distal layer in addition to the beam direction and reached the limit size of 5 mm. These results suggested that the deviation along the beam direction was dominant in the dosimetric error in case that the simple water phantom was assumed for dosimetric evaluation. Note that this is not necessarily applicable to all situations because the WEL could be varied according to the positional deviation along the scanning direction in the actual clinical case.

**Figure 4:**
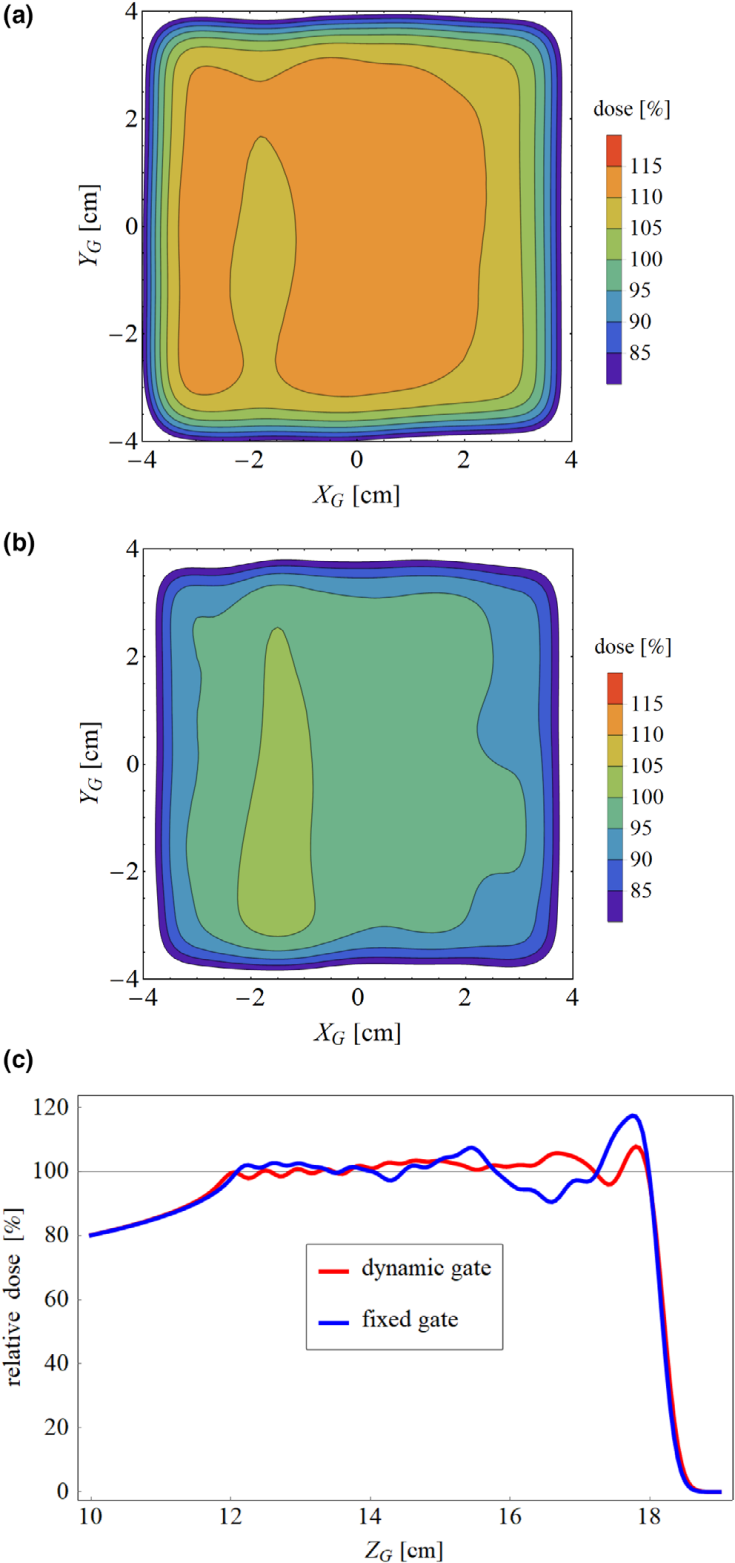
Two‐dimensional dose distribution in the scanning plane of the distal layer in (a) fixed gate and (b) dynamic gate. (c) Dose profile along the beam direction.

**Figure 5:**
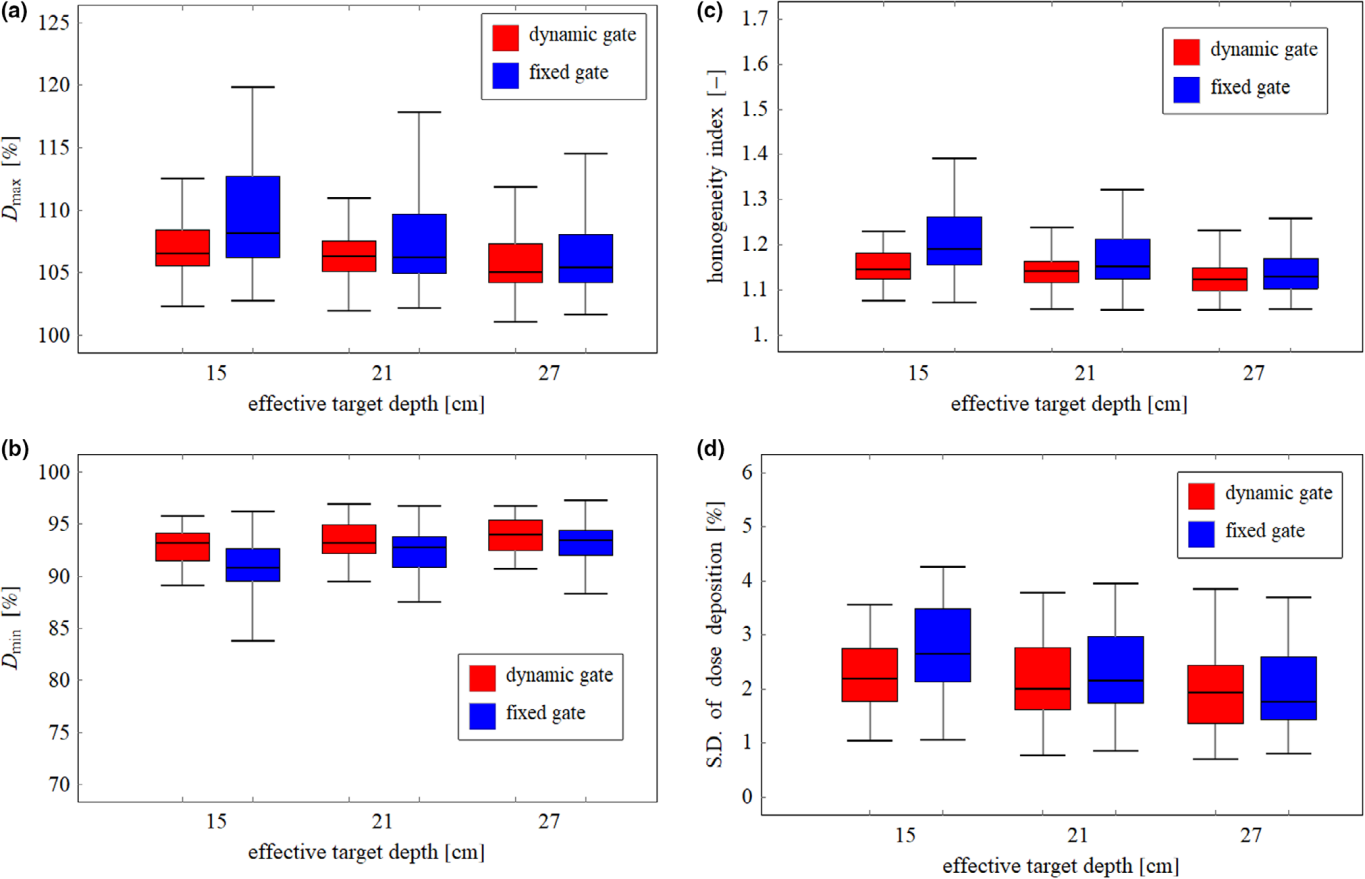
(a) *D*_max_, (b) *D*_min_, (c) HI, and (d) SD in the clinical target volume (CTV) for each effective depth for dynamic and fixed gates.

**Figure 6:**
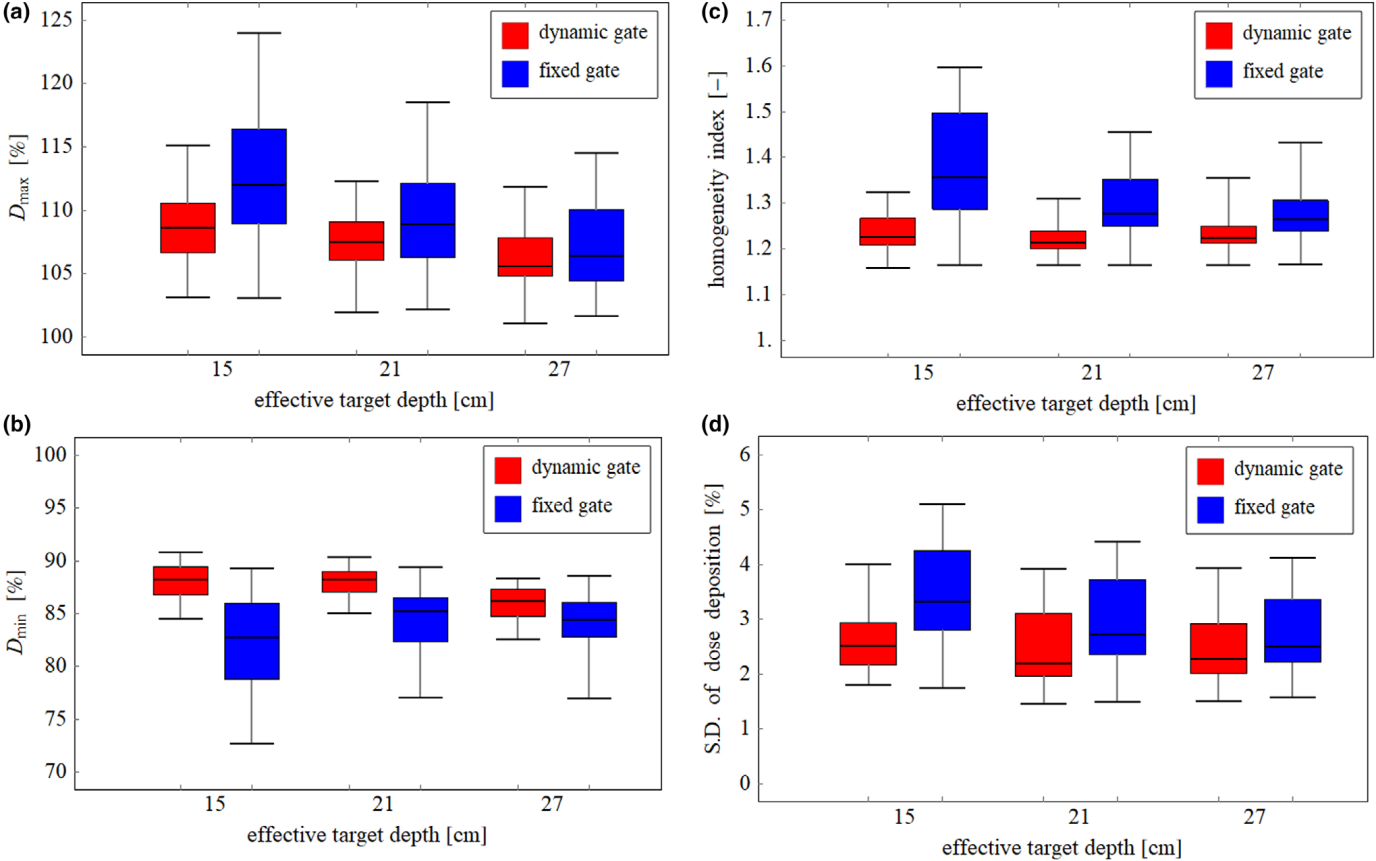
(a) *D*_max_, (b) *D*_min_, (c) HI, and (d) SD in the target volume (TV) for each effective depth in dynamic and fixed gate.

An example of the two‐dimensional dose distribution in the scanning plane at the distal end of the TV with fixed and dynamic gate is shown in Figs. 4(a) and 4(b), respectively. Higher dose than prescribed dose was delivered and shifted laterally overall in fixed gate compared with dynamic gate. The dose profile along the beam direction at the field center is shown in Fig. 4(c). In the fixed gate approach, a large dosimetric error was observed compared with that of the dynamic gate especially in the distal region. In the dynamic gate approach, the dosimetric error was reduced because the smaller size of the gating window was used for the spots with high weight, as shown in Fig. 3. The box plots of *D*_max_, *D*_min_, HI, and SD in the CTV and the TV, which were evaluated for 34 cases, are shown in Figs. 5 and 6, respectively. In both CTV and TV, the dosimetric error in *D*_max_ and *D*_min_ exceeded 5%, which is the tolerance level to determine the size of the gating window in this evaluation, owing to the superposition of the dosimetric error in the scan and beam directions. In dosimetric characteristics comparison, the dynamic gating window technique showed better performance compared with that of the fixed gate in all CTVs and TVs with different effective depths. The dosimetric accuracy in CTVs was improved by applying the dynamic gate, although the improvement was moderate compared with TVs. The results in this study suggest that dose deterioration within the target owing to the interplay effect can be reduced, and the dose to the adjacent organ‐at‐risk can be reduced compared with that of the fixed gate. The variation of dosimetric characteristics as a function of the target depth was small in dynamic gate compared with fixed gate. These results suggested that the dynamic gating window technique can maintain an acceptable dose distribution regardless of the target depth.

### 3.2 Gating efficiency

**Figure 7:**
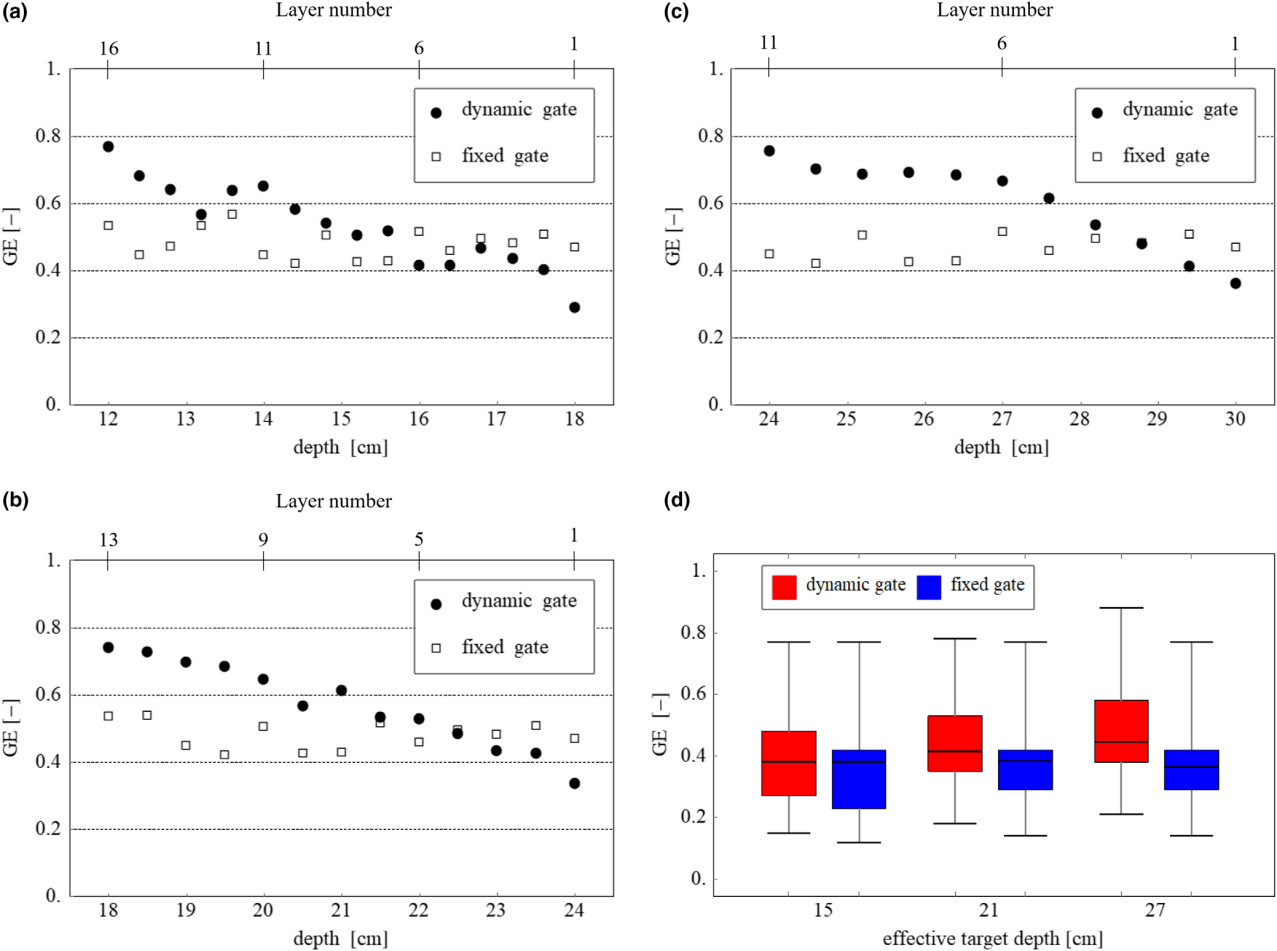
Average gating efficiency (GE) for each layer for dynamic gate and fixed gate at effective depths of (a) 15 cm, (b) 21 cm, and (c) 27 cm. (d) Box plot of the total gating efficiency of 34 cases for dynamic gate and fixed gate for each effective depth.

Gating efficiency was averaged for each irradiation layer, and the irradiation volumes for 34 cases are shown in Fig. 7. As shown in Figs. 7(a)‐7(c), the gating efficiency of the dynamic gate decreased in the distal region because the size of the gating window was reduced to suppress the dose error induced at the spot with high weight. However, in the proximal region, the gating efficiency was increased owing to the enlarged size of the gating window in all directions. Thus, the overall gating efficiency of the dynamic gate was approximately equal or greater than that of the fixed gating window in all evaluated TVs, as shown in Fig. 7(d). For the dynamic gate approach, as the target depth becomes shallower, the gating efficiency is reduced, although the dosimetric characteristics will be maintained regardless of the target depth.

## 4 DISCUSSION

The repaint technique is also effective for treating a mobile target during spot‐scanning.^23^, ^24^, ^25^ Because the dynamic gating window technique can improve the dosimetric accuracy in each painting, better dose distribution can be obtained in less number of repaints by combining the repaint and dynamic gate techniques. Treatment time can be shortened by reducing the repaint number, and it is expected that the imaging dose due to fluoroscopy for real‐time tumor‐tracking can be reduced. Furthermore, the use of novel accelerators with a multiple gating function^26^ will improve the gating efficiency and reduce the treatment time. In this study, we evaluated the gating efficiency instead of the treatment time because the actual treatment time should depend on system specifications and treatment plan parameters such as target size. Because the gating efficiency is increased, the treatment time is expected to decrease.

The main limitation of this study is to assume a simple cubic target for the dosimetric evaluation. In principle, dose characteristics can be improved for the target with an actual shape compared with the fixed gate because the size of the gating window for the spots that may induce large error can be reduced regardless of the target shape. In this case, the maximum dose deviation will not necessarily be observed in the distal region because the spot with a large weight can be irradiated within the target. Because the spot weight varied in a layer, the feasibility of implementing the dynamic gating window function into the treatment system should be discussed. To realize this technique, the size of gating window should be changed in real time during treatment. However, the treatment system that can control the gate size for each spot with sufficient temporal precision during spot irradiation may not be feasible. A possible solution is to optimize the order of spot irradiation to minimize the frequency of change of the gating window size. First, spot positions are sorted according to their optimized gate size. Next, the gate size is rounded into some values. During the treatment, irradiation starts from the spot with a small gate size. Thus, gate size will be increased step by step during irradiation. To modify the gate size during irradiation, it is possible to use the monitoring function of the spot irradiation, which is implemented in a general treatment system. During the treatment planning process, the size of the gating window can be associated with the accumulated monitor units. Although some hardware/software modifications of the treatment system may be required, the monitoring function may be used to hold the beam and to trigger the size change for real‐time imaging equipment with appropriate timing based on the accumulated monitor unit. The typical temporal latency of the treatment system to control the gate size will be acceptable because the spot will be irradiated with reduced gate size even if the control is delayed. Some treatment systems need few seconds to change the energy/layer, and treatment time could be prolonged. In such case, using the same size of gating window which can reduce the dosimetric error in most spots for each layer and varying it from layer to layer could be an alternative method. Because the basic concept and the interface of the gate signal are similar for internal and external surrogates, the proposed technique can be applied in the system based on an external surrogate. However, because the external abdominal and internal tumor motions do not necessarily correlate well during the treatment, it is necessary to confirm their correlation before irradiation.

The other limitation was the optimization method of the gate size for the dynamic gating window technique. In this study, although it was assumed that the displacement of the target position along the beam direction was identical to the WEL variation, it cannot be established in actual clinical practice. The WEL variation owing to respiration in the chest was investigated using 4DCT.^27^, ^28^ It has been reported that the mean intra‐fractional WEL variation for chest wall was less than 4.1 mm for the ITV region. More precisely, the size of the gating window can be optimized by considering the possible dosimetric error using CT images. Currently, it is difficult to evaluate the possible dosimetric error in advance of the treatment with enough temporal and spatial resolution. A novel technique to reconstruct cine‐4DCT[29, 30, 31, 32] with high‐temporal resolution may be applied to determine the gate size by evaluating the actual WEL variation according to the target location.

## 5 CONCLUSION

In this study, the dynamic gating window technique was proposed to improve the dose distribution to treat mobile targets in spot‐scanning particle therapy. The effectiveness of the dynamic gating window technique was validated using a simulation study with a simple phantom geometry because the treatment system that has this function was not available in the current situation. Although a simple cubic target was assumed in the simulation, the results in this study suggest that the proposed gating technique was potentially applicable to improve the dose distribution. The possible solution to implement the dynamic gate function in the treatment system was discussed. When the treatment system with the dynamic gate function is realized, additional evaluations for possible clinical application should be undertaken in the future by accounting for the specifications of the treatment system and clinical situation.

## CONFLICT OF INTEREST

The authors have no conflict of interest to disclose.
